# Coronary Artery Calcium Score and Carotid Intima-Media Thickness As Markers of Computed Tomography Angiographic Severity in Coronary Artery Disease: A Cross-Sectional Study

**DOI:** 10.7759/cureus.113403

**Published:** 2026-07-26

**Authors:** Sachin Khanduri, Tariq Ahmad Imam, Sana, Shaista Ejaz, Deole Yash Milind, Sibtain Raza Khan, Nadeem Akhter, Rubina Saudi, Tanzeel Danish, Punyajeet Sardana

**Affiliations:** 1 Department of Radiodiagnosis, Era's Lucknow Medical College and Hospital, Lucknow, IND; 2 Department of Radiodiagnosis, Era’s Lucknow Medical College and Hospital, Lucknow, IND

**Keywords:** carotid intima-media thickness, coronary artery calcium score, ct coronary angiography, gensini score, syntax score

## Abstract

Introduction: Coronary Artery Calcium Score (CACS) and Carotid Intima-Media Thickness (CIMT) are widely accepted non-invasive indicators of subclinical atherosclerotic burden. Although their prognostic value for predicting cardiovascular events is well established, data linking these markers to the anatomical complexity of coronary artery disease (CAD) - particularly when assessed with computed tomography (CT) derived Synergy Between Percutaneous Coronary Intervention With Taxus and Cardiac Surgery (SYNTAX) and Gensini scoring systems - remain sparse. This study was designed to quantify the relationship between CACS, CIMT and the CT angiographic severity of CAD.

Methods: A cross-sectional study enrolling 75 symptomatic patients referred for CT coronary angiography (CTCA) was conducted over a period of 24 months at a single tertiary centre. CACS was quantified by the Agatston method on non-contrast electrocardiogram-gated CT. CIMT was assessed bilaterally with high-resolution B-mode ultrasonography following the Mannheim consensus. Disease severity was graded using CT-adapted SYNTAX and Gensini scores. Statistical analysis included Pearson's correlation, one-way ANOVA, and chi-square testing (p < 0.05 considered significant).

Results: The cohort comprised 75 patients (mean age 58.84 ± 9.75 years; 62.7% male). Mean CACS was 291.15 ± 164.46 Agatston units and mean CIMT was 0.82 ± 0.18 mm. CACS demonstrated a very strong positive correlation with SYNTAX score (r = 0.813; p < 0.001) and Gensini score (r = 0.851; p < 0.001). CIMT showed a moderate-to-strong positive correlation with SYNTAX score (r = 0.583; p < 0.001) and a strong positive correlation with Gensini score (r = 0.694; p < 0.001). Both CACS and CIMT were significantly associated with severity of coronary artery disease.

Conclusion: CACS and CIMT correlate significantly with CT-derived measures of coronary disease severity, underscoring their complementary roles in non-invasive cardiovascular risk stratification. Combined assessment of these markers may improve patient selection for advanced imaging and individualise preventive management.

## Introduction

Cardiovascular disease continues to be the foremost driver of global mortality, accounting for approximately 17.9 million deaths annually, of which coronary artery disease (CAD) is the single largest contributor [1]. The burden is especially pronounced in South Asia: in India, ischaemic heart disease now accounts for nearly 23% of total deaths and 32% of adult deaths, a proportion that has roughly doubled over the past two decades [2]. A particularly concerning feature is that Indian patients develop symptomatic CAD 5-8 years earlier than Western cohorts, placing a premium on strategies that identify and risk-stratify disease at a subclinical stage [3].

Computed tomography coronary angiography (CTCA) has emerged as a leading non-invasive imaging platform that enables comprehensive anatomical characterisation of the coronary tree-including lumen dimensions, plaque morphology and stenosis severity-without the procedural risks of invasive coronary angiography (ICA) [4]. Quantitative scoring systems originally developed for ICA, namely the Synergy Between Percutaneous Coronary Intervention With Taxus and Cardiac Surgery (SYNTAX) score and the Gensini score, have been adapted for CTCA data with good reproducibility [5,6]. The CT-SYNTAX score grades lesion anatomical complexity (location, bifurcation involvement, total occlusion, calcification, vessel tortuosity) while the CT-Gensini score weighs the degree of luminal narrowing by the functional importance of each coronary segment, together offering complementary dimensions of disease severity [7].

Coronary Artery Calcium Score (CACS), derived from non-contrast cardiac CT using the Agatston algorithm, quantifies the calcified atherosclerotic plaque burden across the coronary vasculature and has demonstrated robust predictive value for major adverse cardiovascular events independent of traditional risk factors [8,9]. Carotid Intima-Media Thickness (CIMT), measured with high-resolution B-mode ultrasonography, reflects early structural changes at the arterial wall driven by endothelial dysfunction and lipid accumulation, and serves as a surrogate for generalised vascular ageing [10,11]. Both markers have been extensively validated as predictors of CAD presence; however, their relationship with the severity and anatomical complexity of CAD-especially when quantified using CT-derived scoring-remains incompletely characterised [12,13].

Several prior studies have reported associations between CACS or CIMT and angiographically confirmed CAD severity, predominantly utilising invasive coronary angiography [14,15]. Studies using CTCA-based severity assessment are comparatively fewer, and simultaneous evaluation of both CACS and CIMT against CT-derived SYNTAX and Gensini scores in the same cohort has rarely been undertaken [16]. Establishing these relationships could extend the clinical utility of CACS and CIMT beyond mere risk prediction toward providing actionable information about the extent and complexity of underlying coronary disease.

The present study aimed to evaluate and compare the correlations of CACS and CIMT with CT-derived SYNTAX and Gensini scores in a cross-sectional cohort of symptomatic patients undergoing CTCA at a single tertiary referral centre.

## Materials and methods

Study design and population

This cross-sectional observational study was conducted from May 10, 2024, to May 10, 2026 (24 months) at the Department of Radiodiagnosis in collaboration with the Department of Cardiology at Era’s Lucknow Medical College and Hospital, Lucknow, Uttar Pradesh, India. Ethical approval was obtained from the Institutional Ethics Committee of Era’s Lucknow Medical College and Hospital, Era University (IRB Approval No. ELMC & H/R-Cell/2024/286, dated May 24, 2024). Symptomatic patients with clinical suspicion of CAD who were referred for CTCA were prospectively enrolled. The sample size was calculated based on a published least correlation coefficient of r = 0.405 between SYNTAX score and CACS/CIMT reported by Pathakota et al. using a formula for correlation studies at α = 0.05 and 90% power, yielding a minimum requirement of 75 participants [14].

Patients were excluded if they had a known contrast allergy, impaired renal function (serum creatinine > 1.5 mg/dL), active pregnancy, haemodynamic instability, significant arrhythmia affecting image quality, congenital heart disease, total CACS > 450 Agatston units (due to bloom artefact degrading CTCA interpretability) or prior coronary revascularisation (CABG or PCI) [17]. Baseline demographic data, anthropometrics, blood pressure, heart rate and cardiovascular risk factors were recorded on a standardised case record form prior to imaging.

Coronary Artery Calcium Score (CACS) measurement

**Figure 1:**
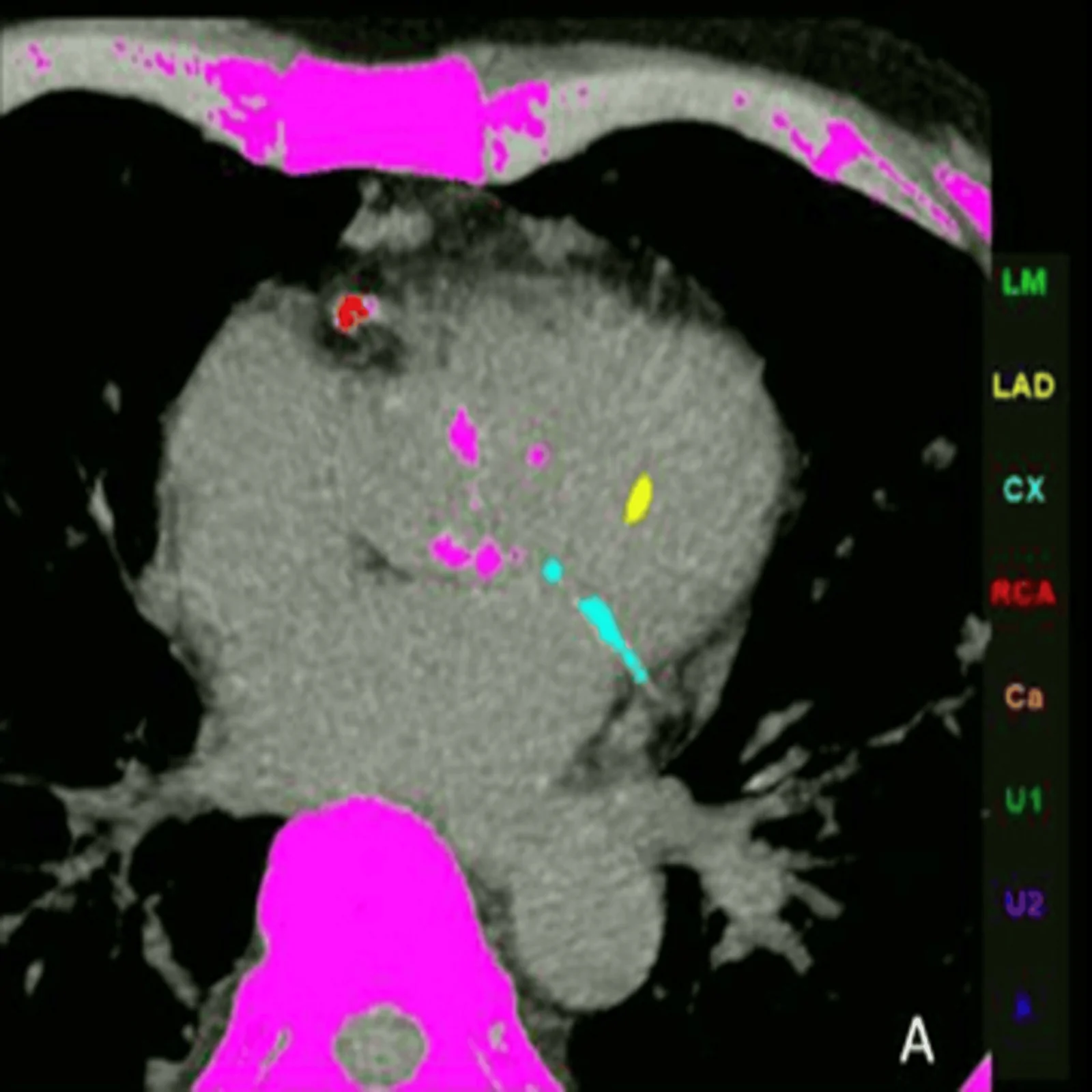
Axial non-contrast cardiac computed tomography demonstrating color-coded calcified plaques in the left anterior descending artery (yellow), left circumflex artery (cyan), and right coronary artery (red) used for Agatston scoring.

**Figure 2:**
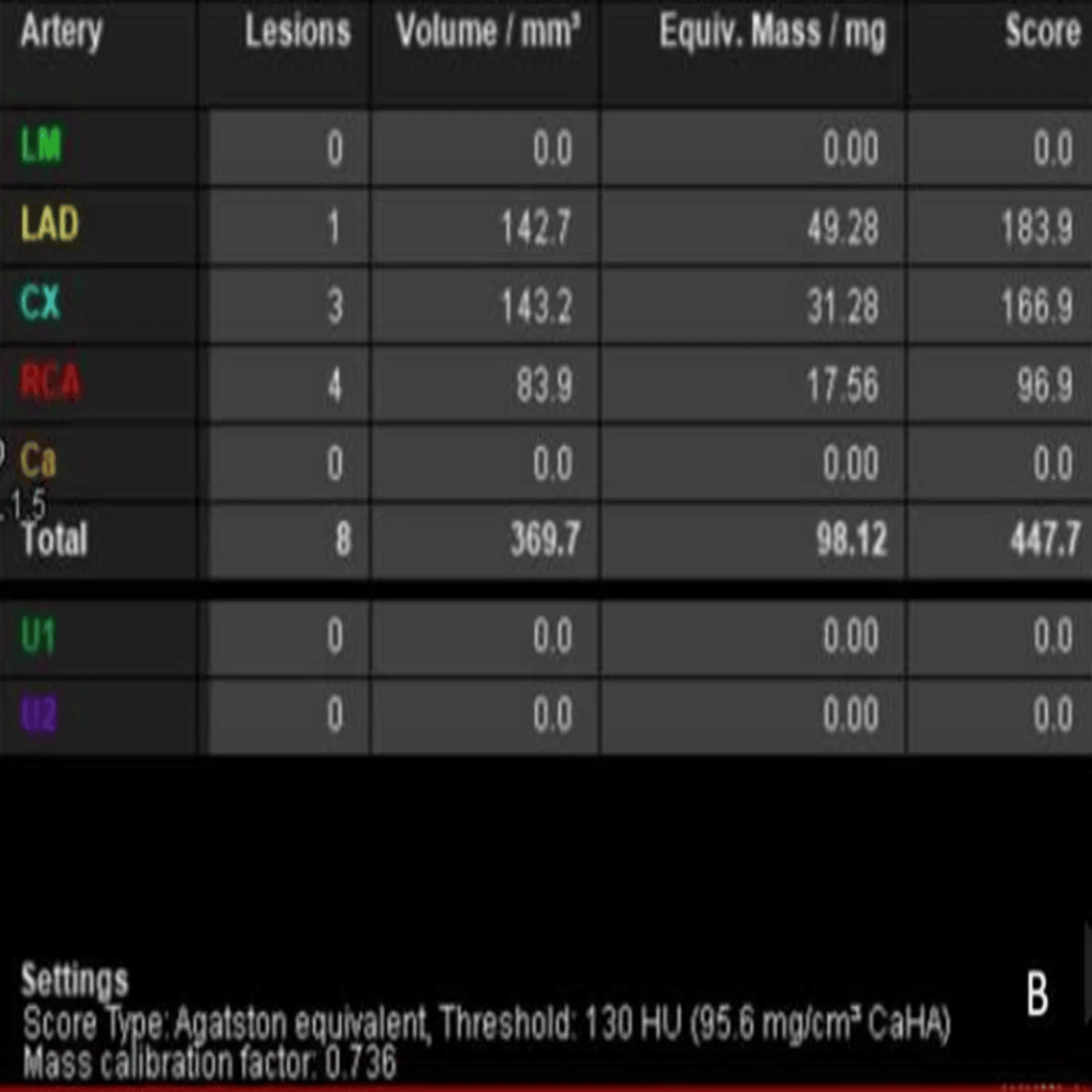
Dedicated software (Syngo Calcium Scoring) report displaying per-vessel lesion count, volume, equivalent mass, and total Agatston CACS (447.7 in this representative case). CACS: Coronary Artery Calcium Score. Syngo Calcium Scoring (Siemens Healthcare GmbH, Erlangen, Bavaria, Germany).

Non-contrast ECG-gated cardiac CT was acquired using a 384-slice dual-source Multi-detector computed tomography scanner (SOMATOM Force, Siemens Healthcare GmbH, Erlangen, Bavaria, Germany) with prospective ECG triggering, 3 mm reconstructed slice thickness and 120 kV tube voltage with automated current modulation. The Agatston scoring method was applied using dedicated software (Syngo Calcium Scoring, Siemens Healthcare GmbH, Erlangen, Bavaria, Germany) [18]. A calcified focus was defined as ≥ 1 mm² area with peak attenuation ≥ 130 HU across at least three contiguous pixels. Each focus was scored by multiplying its area by a density weighting factor (1 for 130-199 HU; 2 for 200-299 HU; 3 for 300-399 HU; 4 for ≥ 400 HU). Individual lesion scores from the left main, LAD, LCX, and RCA were summed to derive the total Agatston score as shown in Figures 1-2. Patients were categorised by CACS as: 0 (absent), 1-99 (mild), 100-399 (moderate), and ≥ 400 (severe) [19].

CT coronary angiography and CAD severity assessment

Contrast-enhanced CTCA was performed using the same scanner following CACS acquisition in patients with CACS ≤ 450. Non-ionic iodinated contrast (iohexol, 350 mg/mL; 1.5 mL/kg at 5 mL/s) was administered with automated bolus tracking (Region of interest at descending aorta; trigger threshold 150 HU). Retrospective ECG gating was applied for heart rates > 70 bpm; prospective gating for stable rates ≤ 70 bpm. Images were reconstructed at 0.6 mm slice thickness and post-processed with multiplanar reformation (MPR), curved planar reformation (CPR), maximum intensity projection (MIP), and volume-rendered technique (VRT) on a Syngo.via workstation (Siemens Healthcare GmbH, Erlangen, Bavaria, Germany) [20].

CT-derived SYNTAX score

**Figure 3:**
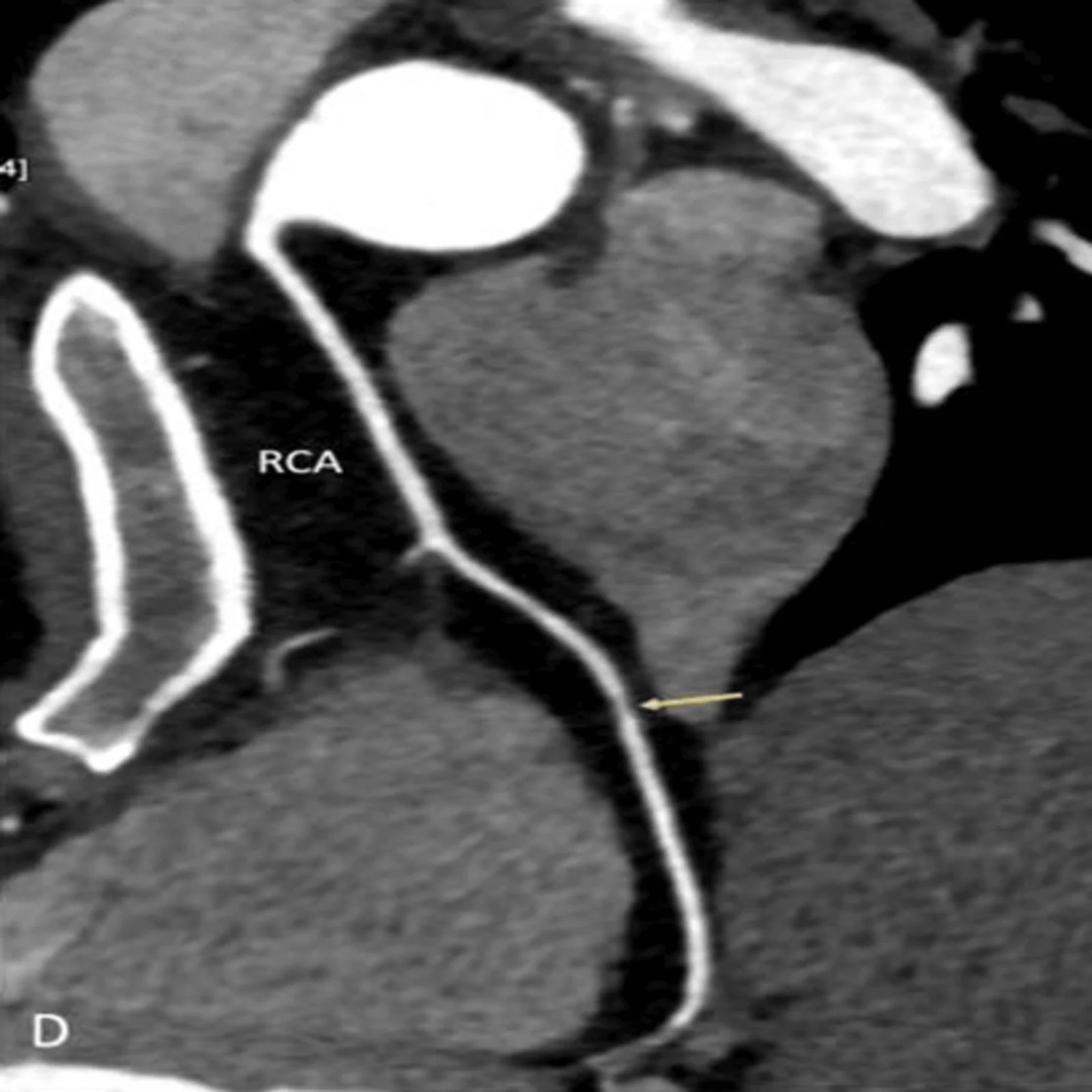
CT coronary angiography (curved planar reformation; Syngo.via) of the right coronary artery (RCA). The arrow marks a significant stenotic segment contributing to elevated Synergy Between Percutaneous Coronary Intervention With Taxus and Cardiac Surgery (SYNTAX) and Gensini scores in this patient. Syngo.via workstation (Siemens Healthcare GmbH, Erlangen, Bavaria, Germany).

The CT-SYNTAX score was computed using the validated online algorithm (www.syntaxscore.org), incorporating all lesions producing ≥ 50% stenosis in vessels ≥ 1.5 mm diameter. Each lesion was graded for anatomical location, bifurcation and trifurcation involvement, total occlusion characteristics, calcification severity, tortuosity, thrombus, and diffuse disease [5]. Scoring was performed by an experienced radiologist blinded to CACS and CIMT values. Patients were stratified by CT-SYNTAX score into: 0 (no significant CAD), 1-22 (low complexity), 23-32 (intermediate complexity), and ≥ 33 (high complexity) categories [7]. A representative example of CT coronary angiography used for SYNTAX scoring is shown in Figure 3.

CT-derived Gensini score

The CT-adapted Gensini score was assigned by grading percentage luminal narrowing per lesion (≤ 25% = 1; 26-50% = 2; 51-75% = 4; 76-90% = 8; 91-99% = 16; total occlusion = 32) and multiplying by the segmental weighting factor reflecting functional significance (left main × 5; proximal LAD/LCX × 2.5; mid-LAD × 1.5; distal LAD, distal LCX, RCA, PDA, obtuse marginals × 1; small branches × 0.5). [6] Weighted lesion scores across all segments were summed to yield the total Gensini score. Gensini score severity categories were divided as : 0 (no significant CAD), 1-10 (mild), 11-38 (moderate), > 38 (severe). A representative example of CT coronary angiography used for Gensini scoring is shown in Figure 3.

Carotid Intima-Media Thickness (CIMT) assessment

**Figure 4:**
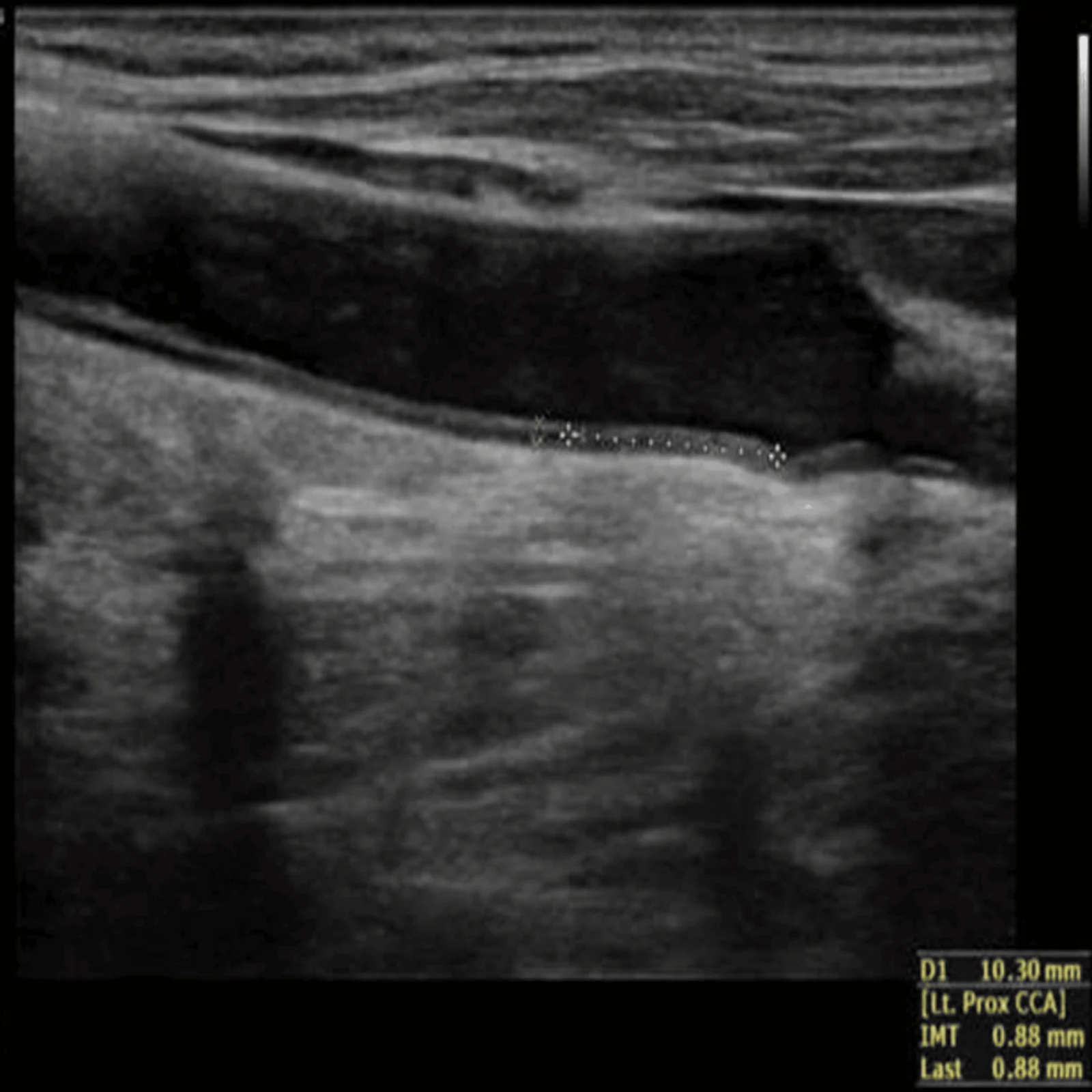
B-mode ultrasound of the left proximal common carotid artery demonstrating far-wall CIMT measurement of 0.88 mm. CIMT: Carotid Intima-Media Thickness.

**Figure 5:**
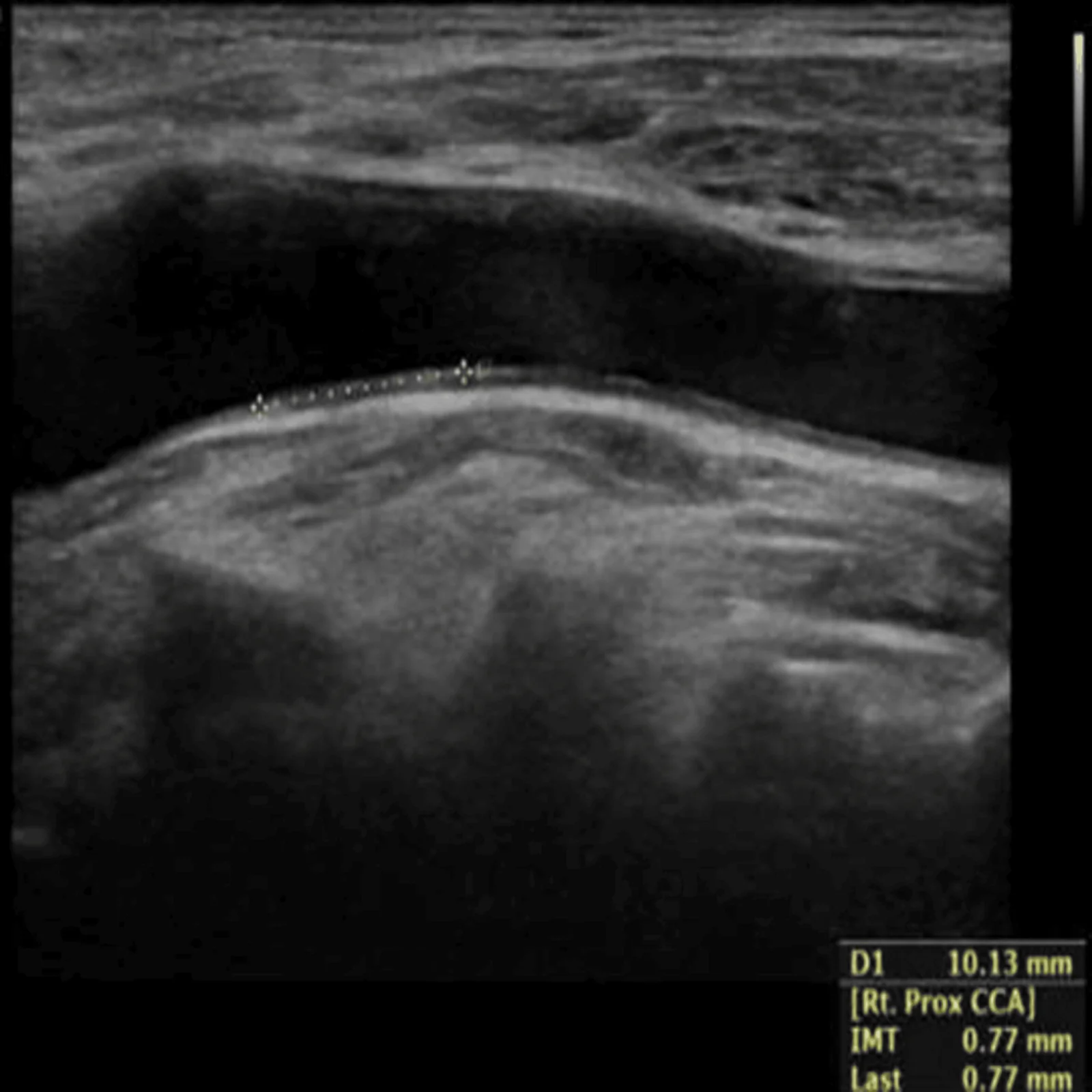
B-mode ultrasound of the right proximal common carotid artery demonstrating far-wall carotid intima media thickness measurement of 0.77 mm.

All enrolled patients underwent bilateral carotid ultrasonography using a Samsung HS70A system (Samsung Medison Co., Ltd., Seoul, Republic of Korea) equipped with a 7-12 MHz high-frequency linear array transducer. Measurements were obtained from the far wall of the distal 1-2 cm segment of each common carotid artery (CCA), approximately 1-2 cm proximal to the carotid bulb, in strict accordance with the Mannheim Carotid Intima-Media Thickness and Plaque Consensus [21]. CIMT was defined as the distance between the lumen-intima and media-adventitia interfaces on end-diastolic images. Three measurements per side were averaged, and the mean of both sides constituted the study value, as shown in Figures 4-5. Focal plaque regions were excluded from measurement. All scans were performed by a single experienced radiologist. Carotid intima-media thickness was categorized as normal (<0.7 mm), borderline (0.7-0.8 mm), or increased (>0.8 mm) [22].

Statistical analysis

Data were captured on a standardised proforma and entered into Microsoft Excel 2023 (Microsoft Corporation, Redmond, WA, USA), then analysed with IBM SPSS Statistics, version 25.0 (IBM Corp., Armonk, NY, USA). Continuous data conforming to normality (Shapiro-Wilk test) are reported as mean ± SD; non-normally distributed variables as median with interquartile range (IQR). Categorical variables are reported as frequencies and percentages. Between-group comparisons for continuous variables were performed using Student's independent t-test (two groups) or one-way ANOVA with Bonferroni post-hoc correction (> two groups). The chi-square test with Monte Carlo correction was applied for categorical associations. Bivariate correlations were assessed using Pearson's r. Statistical significance was set at p < 0.05. Correlation magnitude was interpreted using conventional benchmarks: very weak (r 0.00-0.19), weak (0.20-0.39), moderate (0.40-0.59), strong (0.60-0.79), and very strong (0.80-1.00).

## Results

Baseline characteristics

The study enrolled 75 patients with a mean age of 58.84 ± 9.75 years (range 41-86 years); 47 were male (62.7%). The age distribution was concentrated in the 51-70 year bracket (65.3%). Dyslipidaemia was the most frequent comorbidity (45.3%), with hypertension and diabetes mellitus each present in 38.7%. Tobacco use was reported by 33.3% and alcohol consumption by 30.7%. Mean BMI was 28.48 ± 3.90 kg/m², placing the majority of patients in the overweight category. The full baseline profile is presented in Table 1. Patients harbouring two or more concurrent comorbidities were significantly more likely to have confirmed CAD on CTCA (p = 0.011). Mean BMI was significantly elevated in the CAD group relative to those with normal coronaries (28.80 ± 3.94 vs. 25.44 ± 1.38 kg/m²; p = 0.029).

**Table 1:** Baseline demographic and clinical characteristics of the study population (n = 75).

Variable	Value (n = 75)
Age, mean ± Standard deviation (years)	58.84 ± 9.75
Male sex, n (%)	47 (62.7%)
Body mass index, mean ± Standard deviation (kg/m²)	28.48 ± 3.90
Hypertension, n (%)	29 (38.7%)
Diabetes mellitus, n (%)	29 (38.7%)
Dyslipidaemia, n (%)	34 (45.3%)
Smoking, n (%)	25 (33.3%)
Alcohol use, n (%)	23 (30.7%)
Family history of coronary artery disease, n (%)	5 (6.7%)
Mean coronary artery calcium score (Agatston units), mean ± Standard deviation	291.15 ± 164.46
Mean carotid intima-media thickness (mm), mean ± Standard deviation	0.82 ± 0.18

Distribution of CACS, CIMT, and CAD Extent

Agatston scores spanned 0 to 447.68 (median 381, IQR 145.79-423.26). Severe CACS (≥ 400) was the dominant category (48.0%), followed by moderate scores (30.7%), absent scores (12.0%), and mild scores (9.3%). CIMT values ranged from 0.55 to 1.28 mm (mean 0.82 ± 0.18 mm), with 45.3% exceeding the 0.8 mm threshold. Among the 75 patients, 7 (9.3%) showed no angiographic evidence of CAD, 17 (22.7%) had single vessel disease (SVD), 29 (38.7%) double vessel disease (DVD), and 22 (29.3%) triple vessel disease (TVD). Mean SYNTAX and Gensini scores across the cohort were 16.23 ± 9.74 and 18.80 ± 12.76, respectively.

Association of CACS with CAD severity

**Figure 6:**
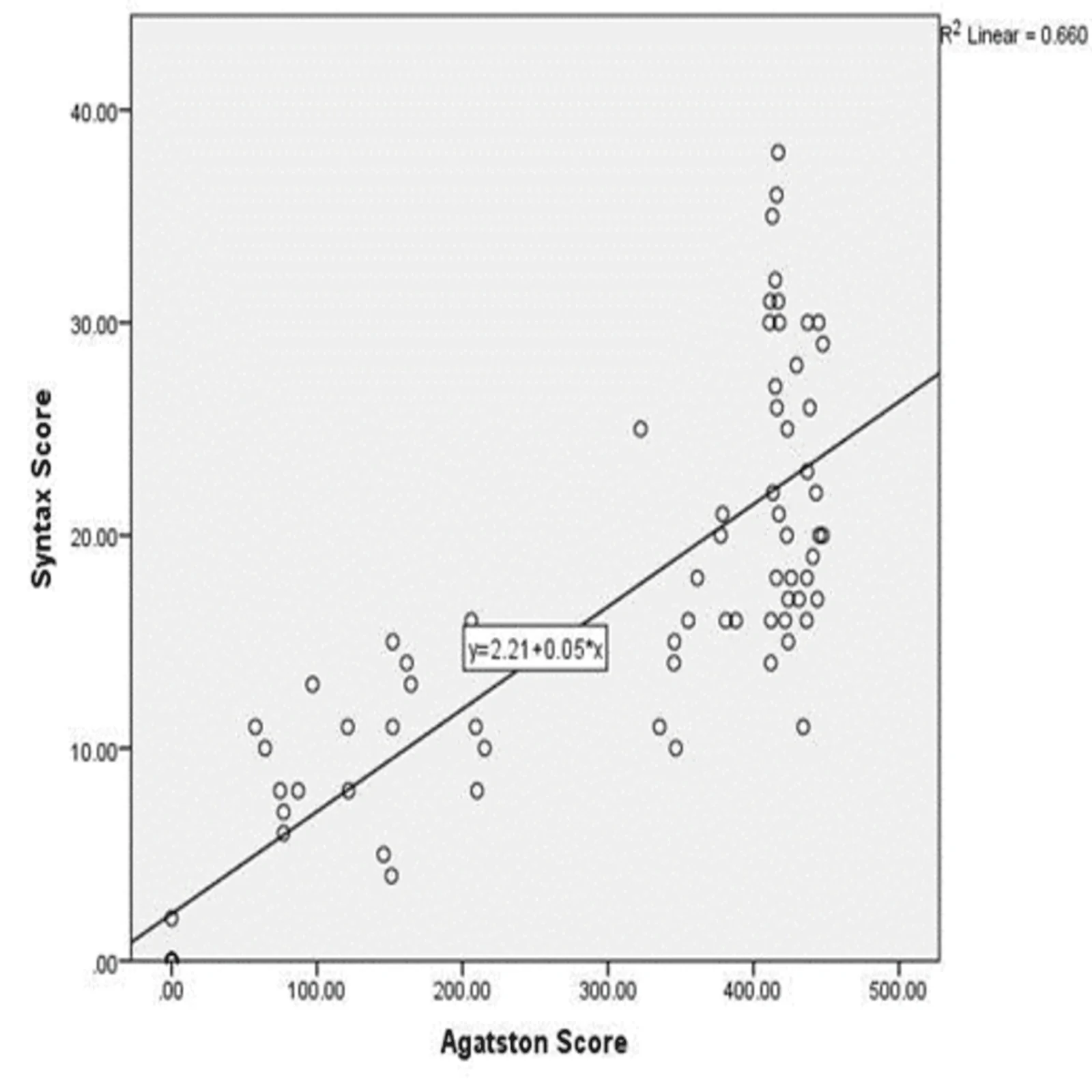
Scatter plot with a fitted simple linear regression line demonstrating the relationship between Agatston CACS on the x-axis and CT-derived SYNTAX score on the y-axis. Pearson correlation analysis showed a strong positive correlation between the two variables (r = 0.813, p < 0.001). Simple linear regression yielded the equation y = 2.21 + 0.05x with a coefficient of determination R² = 0.660 (n = 75). Each circle represents an individual patient. SYNTAX: Synergy Between Percutaneous Coronary Intervention With Taxus and Cardiac Surgery; CACS: Coronary Artery Calcium Score.

**Figure 7:**
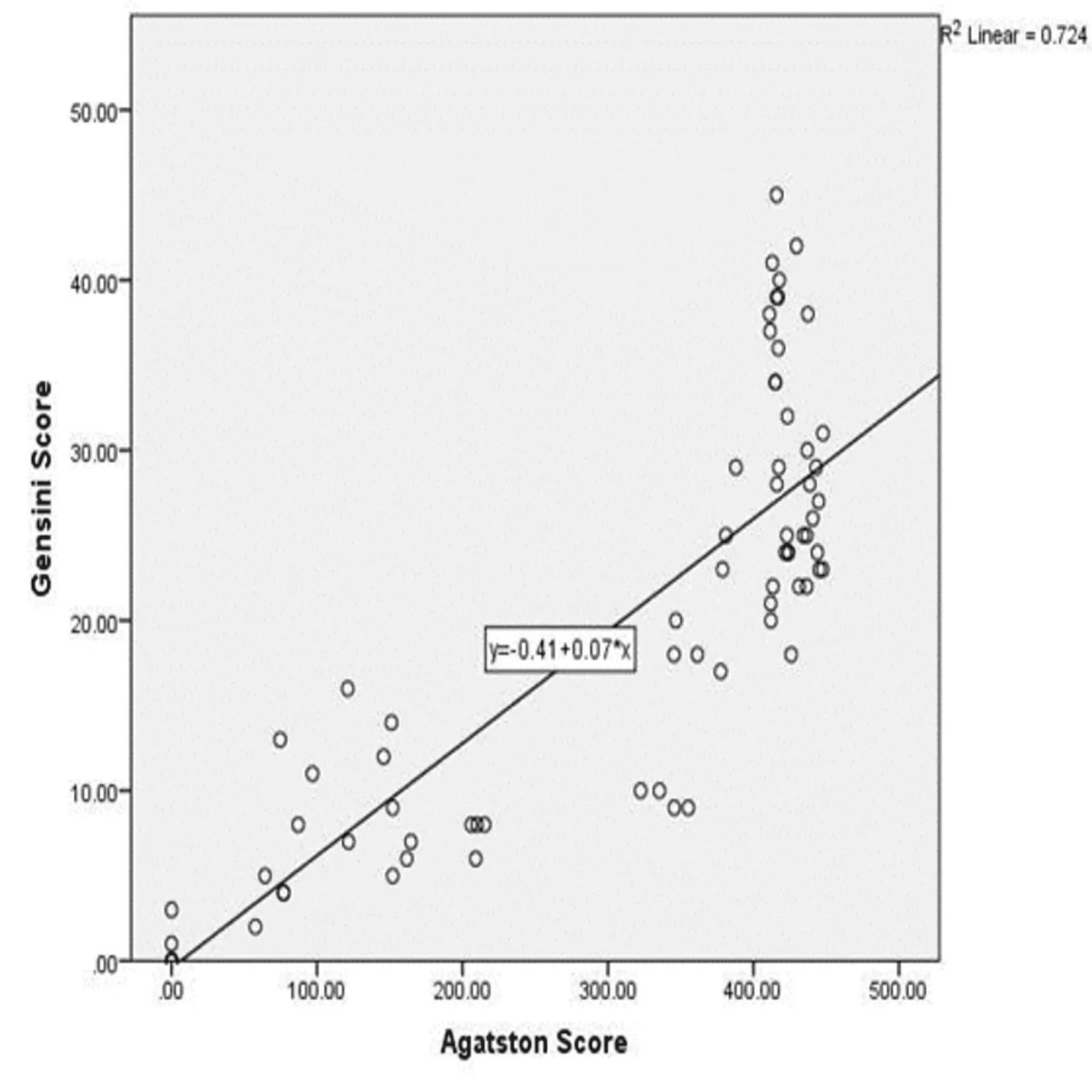
Scatter plot with a fitted simple linear regression line demonstrating the relationship between Agatston CACS on the x-axis and CT-derived Gensini score on the y-axis. Pearson correlation analysis revealed a strong positive correlation between the two variables (r = 0.851, p < 0.001). Simple linear regression yielded the equation y = −0.41 + 0.07x with a coefficient of determination R² = 0.724 (n = 75). Each circle represents an individual patient. CACS: Coronary Artery Calcium Score.

CACS category was significantly associated with both the presence of CAD (χ² = 56.62; p < 0.001) and its extent by vessel involvement (χ² = 41.01; p < 0.001). No patient without CAD had a detectable calcium score, whereas severe CACS (≥ 400) was encountered in 65.5% of DVD patients and 77.3% of those with TVD. Two patients with angiographically confirmed CAD had CACS = 0, consistent with the recognised limitation of non-calcified plaque being invisible to calcium scoring. Correlation analysis demonstrated a very strong positive relationship between CACS and SYNTAX score (r = 0.813; p < 0.001) and between CACS and Gensini score (r = 0.851; p < 0.001). SYNTAX and Gensini scores rose progressively across ascending CACS categories, as displayed in Table 2 and Figures 6-7.

**Table 2:** Comparison of CT-derived SYNTAX and Gensini scores stratified by CACS. Categories (n = 75). Statistical analysis was performed using one-way ANOVA. Values are presented as mean ± standard deviation (SD). SYNTAX: Synergy Between Percutaneous Coronary Intervention With Taxus and Cardiac Surgery; CACS: Coronary Artery Calcium Score.

CACS category (Agatston)	n=75	Mean SYNTAX score ± SD	F-statistic	p-value (one-way ANOVA)	Mean Gensini score ± SD	F-statistic	p-value (one-way ANOVA)
Absent (0)	9	1.22 ± 3.63	F(3,71) = 34.14	< 0.001	1.78 ± 3.35	F(3,71) = 21.84	< 0.001
Mild (1–99)	7	7.57 ± 5.74			8.86 ± 7.56		
Moderate (100–399)	23	14.35 ± 6.40			19.57 ± 8.82		
Severe (≥ 400)	36	24.53 ± 8.12			28.92 ± 12.04		

Association of CIMT with CAD Severity

**Figure 8:**
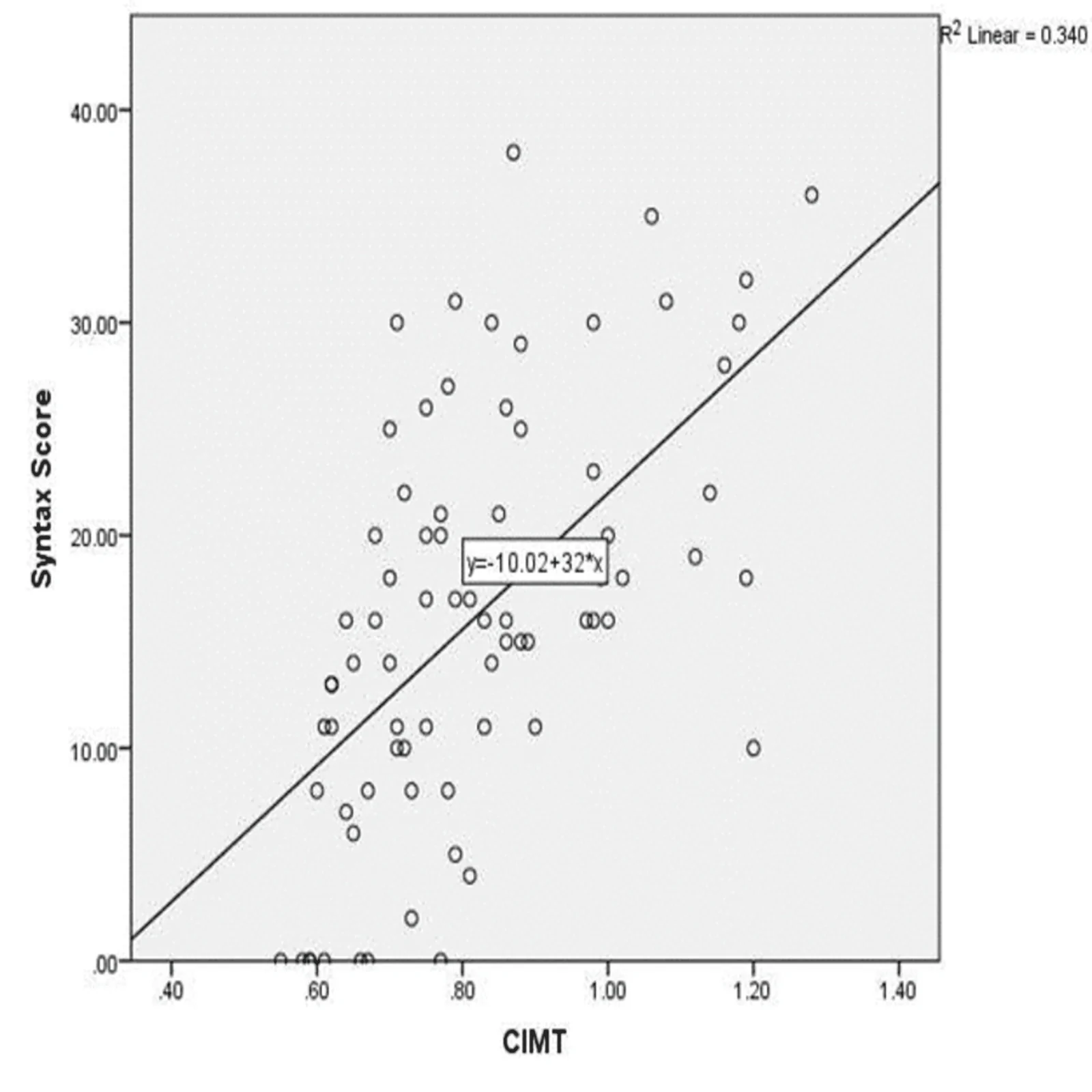
Scatter plot with a fitted simple linear regression line demonstrating the relationship between CIMT (mm) on the x-axis and CT-derived SYNTAX score on the y-axis. Pearson correlation analysis showed a moderate positive correlation between the two variables (r = 0.583, p < 0.001). Simple linear regression yielded the equation y = −10.02 + 32x with a coefficient of determination R² = 0.340 (n = 75). Each circle represents an individual patient. SYNTAX: Synergy Between Percutaneous Coronary Intervention With Taxus and Cardiac Surgery; CIMT: Carotid Intima-Media Thickness.

**Figure 9:**
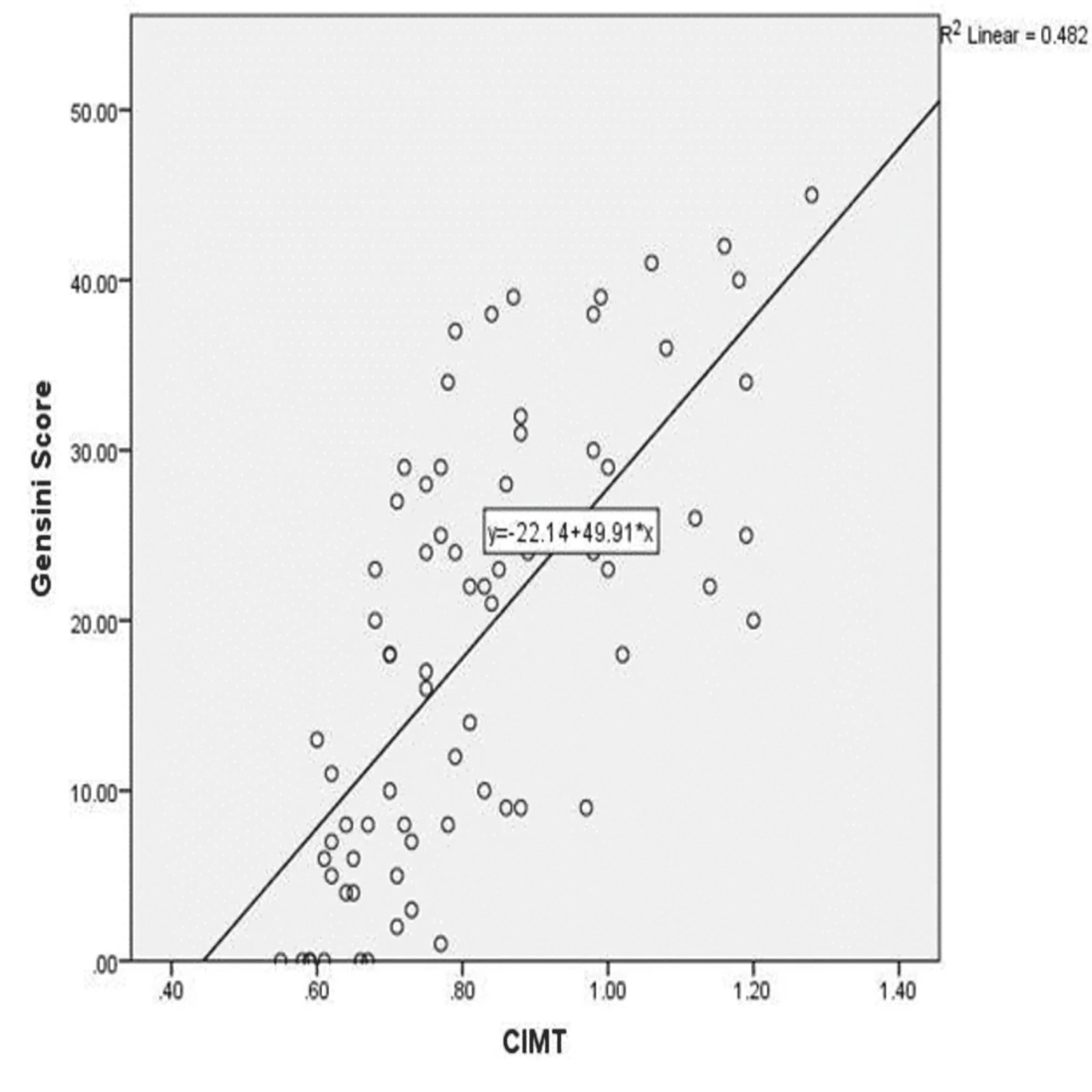
Scatter plot with a fitted simple linear regression line demonstrating the relationship between CIMT (mm) on the x-axis and CT-derived Gensini score on the y-axis. Pearson correlation analysis revealed a moderate-to-strong positive correlation between the two variables (r = 0.694, p < 0.001). Simple linear regression yielded the equation y = −22.14 + 49.91x with a coefficient of determination R² = 0.482 (n = 75). Each circle represents an individual patient. CIMT: Carotid Intima-Media Thickness.

CIMT was significantly associated with both the presence and extent of CAD (χ² = 72.16; p < 0.001). A strict dichotomy was observed at the extremes: all seven patients without CAD had CIMT < 0.7 mm, while all 22 patients with TVD had CIMT > 0.8 mm. Mean SYNTAX score was markedly higher in the CIMT > 0.8 mm group compared with the CIMT ≤ 0.8 mm group (21.21 ± 8.30 vs. 12.10 ± 8.96; t = 4.530; p < 0.001). An analogous gradient was observed for Gensini score (28.85 ± 10.09 vs. 12.12 ± 10.78; t = 6.062; p < 0.001). Table 3 presents these values across CIMT strata. Pearson’s correlation showed CIMT to be moderately-to-strongly associated with SYNTAX score (r = 0.583; p < 0.001) and strongly associated with Gensini score (r = 0.694; p < 0.001) (Figures 8-9).

**Table 3:** Comparison of CT-derived SYNTAX and Gensini scores stratified by CIMT. Categories (n = 75). Statistical analysis was performed using one-way ANOVA. Values are presented as mean ± standard deviation (SD). SYNTAX: Synergy Between Percutaneous Coronary Intervention With Taxus and Cardiac Surgery; CIMT: Carotid Intima-Media Thickness.

CIMT category	n=75	Mean SYNTAX score ± SD	F-statistic	p-value (one-way ANOVA)	Mean Gensini score ± SD	F-statistic	p-value (one-way ANOVA)
< 0.7 mm (normal)	19	6.37 ± 8.02	F(2,72)=22.42	< 0.001	4.47 ± 7.44	F(2,72)=45.13	< 0.001
0.7–0.8 mm (borderline)	22	15.91 ± 6.47			18.55 ± 8.32		
> 0.8 mm (increased)	34	21.21 ± 8.30			28.85 ± 10.09		

Summary of correlation coefficients

A consolidated overview of the Pearson correlation coefficients between the two imaging markers and both CT-derived scoring systems is provided in Table 4. CACS consistently outperformed CIMT in correlation strength with both the SYNTAX and Gensini scores, with CACS-Gensini yielding the highest coefficient of all pairs (r = 0.851).

**Table 4:** Pearson correlation coefficients between CACS/CIMT and CT-derived SYNTAX and Gensini scores. SYNTAX: Synergy Between Percutaneous Coronary Intervention With Taxus and Cardiac Surgery; CACS: Coronary Artery Calcium Score; CIMT: Carotid Intima-Media Thickness.

Imaging marker	Severity score	Pearson r	p-value
Coronary artery calcium score	SYNTAX	0.813	< 0.001
Coronary artery calcium score	Gensini	0.851	< 0.001
Carotid intima media thickness	SYNTAX	0.583	< 0.001
Carotid intima media thickness	Gensini	0.694	< 0.001

## Discussion

The central finding of the present investigation is that both CACS and CIMT are significantly and progressively correlated with CT-derived measures of CAD severity-specifically the CT-SYNTAX and CT-Gensini scores-in a symptomatic single-centre cohort. These findings extend the known prognostic relevance of these non-invasive markers from the prediction of future cardiovascular events to the characterisation of current coronary disease burden [8,11].

The very strong CACS-Gensini correlation (r = 0.851) and CACS-SYNTAX correlation (r = 0.813) observed in the present study are numerically higher than those reported in most prior series using invasive angiography. Pathakota et al. documented a weak-to-moderate correlation (r = 0.405) between CACS and SYNTAX score derived from conventional coronary angiography [14]. Similarly, Gepner et al. reported a positive association between coronary artery calcium score and Gensini score [15]. The superior correlation coefficients in our data likely reflect the methodological advantage of CT-based assessment: the Agatston score and the CT-SYNTAX/Gensini scores are derived from the same imaging modality and the same acquisition, capturing the same coronary territory with the same spatial resolution, thereby minimising inter-modality variability. Furthermore, CT angiography evaluates the entire coronary tree, including non-obstructive disease in segments typically overlooked, which may produce tighter correlations with global calcification burden [4].

The progressive stepwise increase in mean SYNTAX and Gensini scores from the absent-CACS group through to the severe-CACS group is conceptually coherent: calcification accumulates in atherosclerotic plaques that are also more likely to produce haemodynamically significant stenosis [19]. Two patients with confirmed CAD carried a CACS of zero, highlighting the acknowledged limitation that CACS is inherently blind to non-calcified, lipid-rich plaques-which may be the dominant plaque phenotype in younger or diabetic individuals. This observation reinforces the value of CTCA as the definitive test, with CACS serving as an upstream triage tool rather than a replacement for lumenography.

The moderate-to-strong CIMT-SYNTAX correlation (r = 0.583) and strong CIMT-Gensini correlation (r = 0.694) are consistent with the shared pathobiological substrate of carotid and coronary atherosclerosis-common risk factor exposure, endothelial dysfunction, lipid infiltration, and inflammatory activation drive parallel structural changes in both vascular territories [10,22]. The particularly strong CIMT-Gensini association may reflect the fact that the Gensini score emphasises stenosis severity and lesion location rather than anatomical complexity per se, aligning more closely with the diffuse, burden-driven process that also thickens the carotid wall.

The binary segregation of CIMT across the spectrum of CAD extent (all no-CAD patients with CIMT < 0.7 mm; all TVD patients with CIMT > 0.8 mm) is a clinically actionable observation. A CIMT threshold of > 0.8 mm could serve as a practical screening criterion to identify patients at higher probability of multi-vessel disease prior to CTCA referral, potentially improving patient triage in resource-limited settings [21,22]. This finding is concordant with those of Gaibazzi et al., who demonstrated the incremental predictive value of CIMT over Framingham risk scoring for predicting angiographically significant CAD [13].

The complementary roles of CACS and CIMT are highlighted by the differential stage of atherosclerotic disease they capture: CACS reflects calcified, advanced plaque-predominantly in obstructive disease-while CIMT represents earlier, pre-stenotic wall remodelling [12]. Their combined use could therefore provide risk information across the full continuum of disease, from subclinical atherosclerosis to complex multi-vessel CAD.

Limitations

This study has several limitations that should be considered when interpreting the findings. The sample size (n = 75) was calculated based on a specific correlation coefficient and may be insufficient to detect interactions across subgroups. Furthermore, the exclusion of patients with CACS >450 Agatston units may have limited the applicability of our findings to individuals with extensive coronary calcification, as severe calcification can reduce the accuracy of CT coronary angiographic assessment because of blooming artefacts. Single-centre enrolment also limits the external generalisability of the results.

The cross-sectional design does not permit causal inference or assessment of temporal relationships between marker values and clinical outcomes. In addition, CT-derived SYNTAX and Gensini scores, although reproducible, remain dependent on image quality and reader experience, introducing potential observer-dependent variability. Non-calcified and mixed plaque morphology was not systematically quantified, which may underestimate the overall atherosclerotic burden in patients with lower CACS.

Future directions

A larger multicentre prospective study with long-term clinical follow-up is warranted to validate these findings and determine the prognostic utility of combined CACS and CIMT assessment in guiding clinical decision-making and therapeutic strategies.

## Conclusions

This study demonstrates that both Coronary Artery Calcium Score (CACS) and Carotid Intima-Media Thickness (CIMT) are significantly associated with the anatomical severity of coronary artery disease, as quantified by CT-derived SYNTAX and Gensini scoring systems. CACS showed a stronger positive correlation with CAD severity (r = 0.813-0.851), while CIMT also demonstrated significant positive correlations (r = 0.583-0.694), suggesting its potential complementary role in reflecting systemic atherosclerotic burden. The progressive increase in both CACS and CIMT values across the spectrum from no CAD to triple-vessel disease supports their utility as non-invasive markers associated with CAD severity. Future multicentre longitudinal studies are warranted to determine whether integrating CACS and CIMT into clinical decision algorithms improves hard cardiovascular endpoints.
